# Using a hypothetical scenario to assess public preferences for colorectal surveillance following screening-detected, intermediate-risk adenomas: annual home-based stool test vs. triennial colonoscopy

**DOI:** 10.1186/s12876-016-0517-1

**Published:** 2016-09-13

**Authors:** Bernardette Bonello, Alex Ghanouni, Harriet L. Bowyer, Eilidh MacRae, Wendy Atkin, Stephen P. Halloran, Jane Wardle, Christian von Wagner

**Affiliations:** 1 Health Behaviour Research Centre, Department of Epidemiology and Public Health, University College London, 1-19 Torrington Place, London, WC1E 7HB UK; 2 Department of Surgery and Cancer, Imperial College London, London, UK; 3 NHS Bowel Cancer Screening Southern Programme Hub, Guildford, Surrey UK; 4 Royal Surrey County Hospital NHS Foundation Trust & University of Surrey, Guildford, Surrey UK

**Keywords:** Cancer surveillance, Faecal immunochemical test, Colonoscopy, Colorectal cancer, Patient preference, Intermediate-risk adenomas

## Abstract

**Background:**

To assess public preferences for colorectal cancer (CRC) surveillance tests for intermediate-risk adenomas, using a hypothetical scenario.

**Methods:**

Adults aged 45–54 years without CRC were identified from three General Practices in England (two in Cumbria, one in London). A postal survey was carried out during a separate study on preferences for different first-line CRC screening modalities (non- or full-laxative computed tomographic colonography, flexible sigmoidoscopy, or colonoscopy). Individuals were allocated at random to receive a pack containing information on one first-line test, and a paragraph describing CRC surveillance recommendations for people who are diagnosed with intermediate-risk adenomas during screening. All participants received a description of two surveillance options: annual single-sample, home-based stool testing (consistent with Faecal Immunochemical Tests; FIT) or triennial colonoscopy. Invitees were asked to imagine they had been diagnosed with intermediate-risk adenomas, and then complete a questionnaire on their surveillance preferences.

**Results:**

22.1 % (686/3,100) questionnaires were returned. 491 (15.8 %) were eligible for analysis. The majority of participants stated a surveillance preference for the stool test over colonoscopy (60.8 % vs 31.0 %; no preference: 8.1 %; no surveillance: 0.2 %). Women were more likely to prefer the stool test than men (66.7 % vs. 53.6 %; *p* = .011). The primary reason for preferring the stool test was that it would be done more frequently. The main reason to prefer colonoscopy was its superiority at finding polyps.

**Conclusions:**

A majority of participants stated a preference for a surveillance test resembling FIT over colonoscopy. Future research should test whether this translates to greater adherence in a real surveillance setting.

**Trial registration:**

International Standard Randomised Controlled Trial Number registry, ISRCTN85697880, prospectively registered on 25/04/2013

**Electronic supplementary material:**

The online version of this article (doi:10.1186/s12876-016-0517-1) contains supplementary material, which is available to authorized users.

## Background

Since 2006, the population of England aged 60–69 years has been invited to take part in the NHS Bowel Cancer Screening Programme (BCSP), which has since been extended to include people up to the age of 75 years. Eligible individuals are offered a biennial guaiac faecal occult blood test (gFOBt), followed by therapeutic colonoscopy if the initial test result is abnormal. Those who have adenomas detected are often referred for surveillance: Per current BCSP guidelines, standard practice is for individuals who have three or more adenomas (one of which is large; ≥1 cm), or five or more smaller (<1 cm) adenomas to be considered high risk of developing CRC and recommended to undergo a colonoscopy in 12 months, followed by colonoscopies at 3-year intervals. Similarly, individuals who have one or more large (≥1 cm) or 3 or 4 smaller adenomas (<1 cm) are considered to be at ‘intermediate risk’ of developing CRC, and are referred for triennial colonoscopies [1].

Although annual colonoscopy is considered the optimal surveillance test for individuals at high risk of CRC, there are limitations to its use for surveillance of people at intermediate risk. It is resource-intensive and low yield; only approximately 3 % of surveillance colonoscopies detect advanced adenomas in intermediate risk patients [2, 3]. In addition, around 20 % of those at intermediate risk (and even high risk) for CRC do not attend colonoscopic surveillance [4]. Factors associated with non-attendance of screening colonoscopy may include laxative bowel preparation [5], anxiety about the procedure [5], expectations of pain and embarrassment [5, 6], and perceived unpleasantness of the test [7]. Hence, there is interest in the possibility of incorporating alternative tests into surveillance in order to mitigate these issues and improve uptake [8, 9].

One possible option is the faecal immunochemical test for haemoglobin (FIT), which may also be more acceptable to invitees: A previous trial randomised screening-naïve individuals to an offer of first-line screening with either FIT or colonoscopy and reported higher uptake of FIT (34.2 % vs. 24.6 %, respectively) [10]. Similarly, a recent study found that after experiencing both tests, patients at average risk of CRC preferred FIT [11]. Using FIT as the primary surveillance test could also have the advantage of reducing demand on endoscopy services resulting from widespread adoption of population-based CRC screening programmes [12]. However, previous studies on people’s preferences for stool tests (not necessarily FIT) vs. colonoscopy have been in the context of first-line screening [11, 13–17] and to our knowledge, only one has been in the context of surveillance [18]. Furthermore, it is uncertain how FIT performs and compares to colonoscopy for follow-up of patients diagnosed with intermediate risk adenomas, in terms of sensitivity and specificity for advanced adenomas and CRC. A large, pragmatic accuracy and efficacy study (‘FIT for Follow-Up’) is in progress to assess this. Although the accuracy of FIT has been reported to be lower than colonoscopy as a one-off test [19], FIT might perform comparably with triennial colonoscopy when offered on a shorter test interval (annually) and at a low concentration threshold (high FIT sensitivity) [8, 9, 20, 21]. The present study is part of a related strand of research regarding people’s preferences for the respective surveillance tests.

In a previous small-scale, qualitative ‘FIT for Follow-Up’ study we used four focus groups to explore attitudes towards an annual single-sample FIT vs. triennial colonoscopic surveillance of individuals at intermediate risk of CRC. The principal finding was that participants with no experience of colonoscopic surveillance had positive perceptions of FIT [18]. Interestingly, one of the key perceived benefits of annual FIT was the higher frequency of testing; participants believed this might improve detection of advanced lesions. Conversely, participants who preferred triennial colonoscopy often felt that this was the more sensitive test for finding polyps or cancer. However, since the previous study was small-scale, the present study aimed to obtain a more definitive estimate of public preferences for these two potential surveillance tests, in a larger sample of screening- and surveillance-naïve individuals. Each participant was asked to imagine they were at intermediate-risk of CRC, consider the two possible surveillance options, and state an overall preference for one method or another on a questionnaire, along with their underlying reasons using a multiple choice question.

## Methods

### Participants

The recruitment strategy has previously been reported in full [22]. In summary, 3100 screening- and surveillance-naïve individuals aged 45–54 years from three General Practices in England (two in Cumbria; one in London), were invited to participate in a survey. Exclusion criteria were (i) a previous diagnosis of bowel cancer or recent (i.e., in the past 6 months) diagnosis of any cancer; (ii) receiving regular colonoscopies; (iii) learning disability or significant cognitive decline; (iv) clinically judged to be unsuitable to participate (e.g., due to physical or psychological difficulties). Eligibility was determined by systematic database queries and inspection of patient records by their primary care physician.

### Design and measures

This study took place as part of a larger survey that compared people’s willingness to undergo four potential first-line screening tests [22]. Eligible individuals were assigned at random by household to receive a study pack, containing a cover letter, a 12-page, double-sided A5 information booklet (‘The Facts’ booklet) and a three-fold, double-sided A4 leaflet (‘Test Information’ leaflet). The cover letter asked invitees to read through the information booklet and leaflet. The Facts booklet explained:

- The concept of screening (i.e., primarily prevention of CRC through detection of adenomas which could become cancers over time but could be removed by prophylactic intervention)
- A summary of how one of four first-line screening tests (colonoscopy, flexible sigmoidoscopy, non-laxative computed tomographic colonography [CTC], or full-laxative CTC) would be carried out
- Possible side-effects and complications from the test
- The chances of possible results and how they would be disseminated
- Recommended follow-up testing where applicable (i.e., colonoscopy following an abnormal flexible sigmoidoscopy or CTC result)
- Sensitivity of the first-line screening test (“over 90 %” for colonoscopy and full-laxative CTC; “over 85 %” for non-laxative CTC; “over 65 %” for flexible sigmoidoscopy)
- Treatment options if cancer is detected.

The Test Information leaflet contained a more detailed explanation of how the first-line screening test would be carried out, including preparation requirements, what would happen at the hospital, any medication that would be required (e.g., sedation in the case of colonoscopy, in accordance with typical practice in the BCSP in England), test duration, and possible after-effects.

#### Information on CRC surveillance

Study packs also included a standardised questionnaire, which gave participants a summary of how and why the results of a colonoscopy could lead to a recommendation of surveillance, and information that possible surveillance tests consisted of triennial colonoscopy (previously described in the information booklets and applicable leaflet) and a stool test resembling FIT (see Additional file 1):

*“Remember that if any polyps were found during the [first line screening test], people might be offered a colonoscopy. A colonoscopy looks at the large bowel using a tiny camera passed through the back passage, and requires a laxative preparation. If people have one large or several small polyps removed during colonoscopy, they would be considered to be at ‘intermediate risk’ of developing bowel cancer. People at intermediate risk would be offered a follow-up test in the future to check that no new polyps have grown. This is called ‘polyp surveillance’. There are several possible follow-up tests.”*

Immediately after the information about surveillance, participants were asked to imagine that they were found to be at intermediate-risk for CRC during their initial screening episode and asked a hypothetical question:

*‘Imagine that you had a large polyp removed during a colonoscopy and were at intermediate risk of developing bowel cancer. If you were offered follow-up testing to check that no new polyps have grown, which option would you prefer?’*

Response options consisted of:

A. ‘Another colonoscopy test every 3 years’
B. ‘A stool test at home every year. This would require me to take a sample from one of my bowel motions and post it back to a laboratory in a hygienically sealed envelope. If this test result were abnormal, I would be offered a colonoscopy.’
C. ‘No follow-up testing’
D. ‘No preference for any of these options’

Option B was designed to resemble the form of stool testing that would be offered to surveillance patients (i.e., FIT, requiring only one stool sample, as distinct from the current gFOBT programme which requires three samples). If participants stated a preference (i.e., they had chosen either option A or B), they were asked to select the reason(s) for their preference from the following list of options and they could choose more than one option: ‘more frequent’; ‘more thorough’; ‘less likely to cause side-effects’; ‘more familiar to me as I (would) have had the test in the past’; ‘better at finding polyps or cancer’; ‘less likely to harm me’; ‘more convenient’; or ‘better at showing me what is happening during the test’. There was also an ‘other (please state)’ option. Since no validated measure of surveillance preferences could be located, items were designed by members of the study team (AG, HB & CVW; see Additional file 2). Response options were based on recurring themes in the previous qualitative study [18].

Participants were asked to report their gender, age, ethnicity, current employment status, and highest level of education achieved. Postcodes were used to calculate 2010 Index of Multiple Deprivation (IMD) scores (an area-based measure of socioeconomic deprivation) [23]. Participants were also asked whether they knew anyone who had been diagnosed with bowel cancer and whether they had ever had a bowel test themselves (i.e., not limited to screening but any bowel test for any purpose).

Participants were asked to complete the questionnaire and return it in a provided Freepost envelope (which constituted consent), or to return the questionnaire blank if they did not wish to participate. After 2 weeks, a reminder letter and a replacement Freepost envelope was issued to all non-responders. All study materials are available in online appendices [22].

### Analysis

Responses were pooled across the four randomised first-line screening test conditions (since surveillance would be recommended following colonoscopy and the closure of a screening episode, irrespective of the pathway to that point). Stated surveillance preferences and underlying reasons were summarised using descriptive statistics. Further analyses tested for differences in preferences between subgroups (e.g., men and women) using Pearson’s χ^2^ tests for association. Fisher’s exact test was used to compare preferences by employment status and education due to expected counts being less than five in some cells. Wilson score 95 % confidence intervals (CI) were calculated for overall preferences, preferences within each subgroup, and reasons for preferences. Responses to the measure of education were divided into three groups: participants with a degree or higher degree were categorised as “high education”, those with a lack of formal qualifications were categorised as “low education” and participants responding in other ways (e.g., A-levels or BTECs) were categorised as “medium education”.

## Results

Out of 3100 invitations, 686 questionnaires were returned across the four conditions (22.1 %; n per condition: 164 to 178), of which 603 participants (19.5 %) were eligible for the main analysis outlined elsewhere [24]. A further 28 (0.9 %) participants who did not answer the question on surveillance preferences and 84 (2.7 %) patients with previous bowel test experience were excluded as the numbers were too small for subgroup analyses, resulting in a final sample of 491 (15.8 %). Demographic characteristics of the sample are shown in Table 1. The median age was 50 years (interquartile range [IQR]: 47 to 52) and median IMD score was 11.4 (IQR: 8.6 to 14.0). Females were more likely to respond to the questionnaire than males (25.8 % vs. 18.8 %), as were white British individuals compared with those from other ethnic groups (40.4 % vs. 13.4 %).Compared with non-responders, responders were also slightly older (median age: 50 vs. 49 years) and living in less deprived areas (median IMD score: 11.5 vs 14.4; all *p*-values < .0005).

**Table 1:** Demographic statistics of respondents with a surveillance preference using a hypothetical scenario of being at intermediate risk for CRC (*n* = 490)

	All participants	Prefer colonoscopy		Prefer a home-based stool test^a^		No preference		*p*-value
	(*n* = 490)^b^	% (*n* = 152)	[95 % CI]	% (*n* = 298)	[95 % CI]	% (*n* = 40)	[95 % CI]	
Gender								.011
Male	(*n* = 220)	35.9 (79)	[29.7, 42.4]	53.6 (118)	[47.0, 60.1]	10.5 (23)	[7.1, 15.2]	
Female	(*n* = 270)	27.0 (73)	[22.1, 32.6]	66.7 (180)	[60.8, 72.0]	6.3 (17)	[4.0, 9.9]	
Ethnicity								.151
White-British	(*n* = 432)	29.4 (127)	[25.3, 33.9]	62.0 (268)	[57.4, 66.5]	8.6 (37)	[6.3, 11.6]	
Non-White British	(*n* = 49)	42.9 (21)	[30.0, 56.7]	51.0 (25)	[37.5, 64.4]	6.1 (3)	[2.1, 16.5]	
Employment status								.960
Employed	(*n* = 426)	31.7 (135)	[27.5, 36.3]	60.3 (257)	[55.6, 64.9]	8.0 (34)	[5.8, 10.9]	
Not employed/retired	(*n* = 34)	29.4 (10)	[16.8, 46.2]	64.7 (22)	[47.9, 78.5]	5.9 (2)	[1.6, 19.1]	
Highest level of education								.064
High education	(*n* = 151)	27.8 (42)	[21.3, 35.4]	61.6 (93)	[53.6, 69.0]	10.6 (16)	[6.6, 16.5]	
Medium education	(*n* = 234)	27.8 (65)	[22.4, 33.8]	64.5 (151)	[58.2, 70.4]	7.7 (18)	[4.9, 11.8]	
Low education	(*n* = 24)	54.2 (13)	[35.1, 72.1]	45.8 (11)	[27.9, 64.9]	0.0 (0)	[0.0, 13.8]	
Indirect CRC experience								.477
Knows someone with bowel cancer	(*n* = 247)	29.6 (73)	[24.2, 35.5]	63.2 (156)	[57.0, 68.9]	7.3 (18)	[4.7, 11.2]	
Does not know someone with bowel cancer	(*n* = 234)	32.5 (76)	[26.8, 38.7]	58.1 (136)	[51.7, 64.3]	9.4 (22)	[6.3, 13.8]	

^a^The home-based stool test was comparable to FIT

^b^n varies because of missing data

Only one participant in the final sample preferred no follow-up testing (0.2 %) and so this category was omitted from subsequent analyses. A small proportion of participants had no preference between the options (*n* = 40; 8.1 %; 95 % CI [6.1 %, 10.9 %]). The majority stated a preference for surveillance with the stool test (*n* = 298; 60.8 %; 95 % CI [56.4 %, 65.0 %]) with colonoscopy preferred less frequently (*n* = 152; 31.0 %; [27.1 %, 35.3 %]). We also found evidence of gender differences in surveillance preferences with women more likely to prefer the stool test than men (66.7 % vs. 53.6 %, χ^2^ [2, *N* = 490] = 9.028, *p* = .011). We did not find evidence to suggest differences in surveillance preferences by ethnicity, employment status, level of education, or indirect experience of CRC (all *p*-values > .05).

The majority of the participants who stated a preference for surveillance with the stool test stated that they did so because they would be tested more frequently (62.1 %) and the test was more convenient (51.7 %; Table 2). A large proportion also said they believed that it was less likely to cause side effects (39.9 %) and less likely to cause harm (32.6 %). The majority of participants who stated a preference for colonoscopy said that they believed the test was better at finding polyps or cancer (77.6 %) and also more thorough (53.9 %). Almost one in three who preferred a colonoscopy gave the reason that they would have already had one and so would be more familiar with the test (29.6 %; Table 2).

**Table 2:** Reasons for surveillance test preferences using a hypothetical scenario of being at intermediate risk for CRC

	Preferred a home-based stool test^a^		Preferred colonoscopy	
	% (*n* = 298)	[95 % CI]	% (*n* = 152)	[95 % CI]
Reason for preference^b^				
More frequent	62.1 (185)	[56.5, 67.4]	21.7 (33)	[15.9, 28.9]
More convenient	51.7 (154)	[46.0, 57.3]	12.5 (19)	[8.2, 18.7]
Less likely to cause side-effects	39.9 (119)	[34.5, 45.6]	7.2 (11)	[4.1, 12.5]
Less likely to harm me	32.6 (97)	[27.5, 38.1]	5.9 (9)	[3.2, 10.9]
Better at finding polyps or cancer	28.9 (86)	[24.0, 34.3]	77.6 (118)	[70.4, 83.5]
More thorough	21.5 (64)	[17.2, 26.5]	53.9 (82)	[46.0, 61.7]
More familiar as I would have had the test before	16.8 (50)	[13.0, 21.4]	29.6 (45)	[22.9, 37.3]
Better at showing what is happening during the test	6.4 (19)	[4.1, 9.8]	17.8 (27)	[12.5, 24.6]
Other reasons	7.0 (21)	[4.5, 10.7]	3.3 (5)	[1.4, 7.5]

^a^The home-based stool test was comparable to FIT

^b^ *N.B* participants could select more than one reason

## Discussion

This study assessed public preferences for two viable surveillance tests that could be offered to patients at intermediate-risk of CRC. We found that the majority of participants stated a preference for surveillance with an annual, single-sample, home-based stool test consistent with FIT over triennial colonoscopy. The findings suggest that participants prioritised frequency, convenience, and lower risk of harm or side effects as the reason for their preference for FIT, and test sensitivity and thoroughness as their reasons for preferring colonoscopy. The views of participants who favoured colonoscopy were also influenced by the perspective that they would have already had a colonoscopy as part of the initial screening pathway. These findings are largely consistent with our previous qualitative study comparing attitudes towards an annual FIT versus triennial colonoscopic surveillance among individuals at intermediate risk of CRC [18]. Stated reasons for participants’ preferences were also similar to those of a study in which participants preferred to be screened using FIT when considering test preparation and avoiding complications but preferred colonoscopy when considering accuracy [11]. It was more surprising that 16.8 % of participants who stated a preference for FIT did so on the basis that they would have had the test before; this may have been related to awareness that the current BCSP is based on first-line stool testing (which they expect to take up in future) and that stool tests are available in other contexts (e.g., for diagnosis).

There were no differences by ethnicity, employment status, education level, or for participants that knew someone who had bowel cancer but women were more likely than men to prefer the stool test over colonoscopy. This latter observation is in line with previous research showing that women have lower attendance rates for screening colonoscopy than men [24, 25], that they hold more negative expectations about the test (e.g., relating to pain or fear of a cancer diagnosis) [25, 26], and are more concerned about modesty, embarrassment, and risks related to the procedure [25, 26]. In terms of colonoscopy experience, women report poorer tolerance [27], more pain [28], and more discomfort [29] during colonoscopy than men, suggesting that some of their pre-existing concerns are well-founded. The higher proportion of women stating a preference for the stool test is also consistent with a meta-analysis of studies on FIT in the context of screening and another recent study that tested patients’ preferences after experiencing both colonoscopy and FIT [11, 30].

As with the previous qualitative study, these findings are most directly applicable to the English BCSP. In organised CRC screening programmes, it is often necessary to offer a default first-line and therapeutic test (e.g., first-line gFOBt and therapeutic colonoscopy) and impractical to offer a range of options. This also applies to surveillance tests when they are offered to a large number of people in a centralised fashion. Previous studies have reported several barriers to colonoscopy attendance [5–7, 24–26] and our findings suggest that a home-based stool test may be a more attractive alternative to colonoscopy. Hence, the present study adds evidence that offering annual FIT as the default surveillance method could be an effective strategy for increasing participation rates in the context of surveillance. Furthermore, it could also lower colonoscopy demand, which is a significant challenge for healthcare systems in several countries [12, 19]. In principle, the BCSP could use a similar system for surveillance of people at intermediate risk of CRC to that used for coordinating first-line screening with gFOBt. Annual, single-sample FIT kits could be posted to eligible individuals, who could then complete the test at home and return it to a laboratory for analysis. However, the preference for a stool test resembling FIT was not universal, with about 1 in 3 participants preferring triennial colonoscopies. One potential method of optimising uptake of CRC surveillance would be to offer FIT as a default test but for colonoscopic surveillance to remain an option for those who persistently decline FIT. These findings also suggest that in healthcare systems that offer a choice of tests, it would be effective to allow patients to select their preferred method.

These possible applications should be considered in the context of current uncertainties regarding the relative sensitivity of annual FIT and triennial colonoscopy. Higher uptake of FIT may not translate to superior health outcomes or cost-effectiveness unless it delivers adequate sensitivity and specificity. The results of the FIT for Follow-Up study will address this important question [8].

An important limitation of this study was that participants were asked to imagine that they were being offered follow-up testing after being diagnosed as intermediate risk of CRC. Therefore, preferences may not be representative of individuals in a real life setting (i.e., with people who had undergone CRC screening, including a diagnostic colonoscopy). For example, one previous study found that a majority of patients undergoing colonoscopy reported that the experience was better than expected [31]. Similarly, patients facing the prospect of surveillance following screening would be older than this sample (and would differ on related demographic characteristics e.g., employment and personal health status). Participants in this study were recruited on the basis of being screening-naïve but approaching the eligible screening age, in order to assess preferences for first-line tests without their perceptions being affected by previous invitations and experience of tests as part of the existing BCSP [22]. However, surveillance preferences may differ to this screening-naïve sample. In addition, although the sample was larger than the previous qualitative study, the response rate was lower than expected, introducing a possible selection bias. Although we observed few differences in preferences between subgroups, the number of participants that were non-white British, not employed or retired, and had a low level of education was lower than in the general population and there may have been differences that could not be detected in this study.

Another important limitation was that participants were only given information about the sensitivity of colonoscopy and not the stool test. In the absence of more definitive estimates, information on the sensitivity of FIT could have been provided using data on its performance as a one-off, first-line test [12] or a biennial test [32]. Estimates of sensitivity for FIT are relatively low compared with colonoscopy in this context. However, they were omitted due to being unlikely to offer a valid approximation of the performance of FIT as a surveillance test repeated over three rounds. Consequently, participants’ perceptions of the stool test may have been based primarily on its appearance (e.g., a non-invasive and less thorough test may have also been perceived as less capable of detecting adenomas) and preferences may change in light of additional information [11]. Future research could determine whether preferences are consistent when people are also given this information, once more applicable estimates are available.

Furthermore, the four randomised conditions provided slightly different information about the first-line screening tests. For example, invitees allocated to receive literature about colonoscopy for first-line screening were provided with extra information about the test e.g., that it was *‘over 90 % accurate for detecting polyps and cancer’*, whereas invitees in other conditions were given the associated statistic for a different test (either flexible sigmoidoscopy or a form of CTC). This meant that participants receiving information about colonoscopy might have had more knowledge about its capabilities, particularly since information on accuracy of FIT as a diagnostic test was not provided. However, although information and measures relating to preferences for first-line screening tests may have been distracting, when we compared surveillance preferences across the four conditions we did not find statistically significant differences. The numbers were small but this suggests that the type of information participants received regarding screening did not affect their preferences for surveillance tests.

## Conclusions

When asked to imagine being at intermediate risk of CRC, individuals aged 45–54 years generally stated a preference for an annual single-sample home-based stool test resembling FIT over triennial colonoscopy for surveillance. FIT may be a viable alternative to colonoscopy, and may be favoured by women in particular. Future research should consider whether this preference translates to improved adherence in a real surveillance setting.

## Additional files

Additional file 1: Information provided on the two surveillance tests. (DOCX 15 kb) Additional file 2: Questionnaire items relating to surveillance. (DOCX 1099 kb)

## Abbreviations

BCSP: Bowel cancer screening programme
CI: Confidence interval
CRC: Colorectal cancer
CTC: Computed tomographic colonography
FIT: Faecal immunochemical test
gFOBT: Guaiac faecal occult blood test
IMD: Index of multiple deprivation
IQR: Interquartile range
