# Supplementary material for: Using a hypothetical scenario to assess public preferences for colorectal surveillance following screening-detected, intermediate-risk adenomas: annual home-based stool test vs. triennial colonoscopy
Source: BMC Gastroenterol. 2016 Sep 13;16(1):113. doi: 10.1186/s12876-016-0517-1 (PMC5020544; doi:10.1186/s12876-016-0517-1)
Supplement: Additional file 1: — Information provided on the two surveillance tests. (DOCX 15 kb) [file 12876_2016_517_MOESM1_ESM.docx]

# Additional file 1 - Information provided on the two surveillance tests

|  |  |  | | | |
| --- | --- | --- | --- | --- | --- |
|  | **Information on colonoscopy** | | **Information on the home-based stool test** | | |
|  |  | |  | | |
| **Received by all participants via the questionnaire:** | | | | | |
|  | . Remember that if any polyps were found during the [first-line screening] test, people might be offered a colonoscopy. A colonoscopy looks at the large bowel using a tiny camera passed through the back passage, and requires a laxative preparation. | | | . A stool test at home every year. This would require me to take a sample from one of my bowel motions and post it back to a laboratory in a hygienically sealed envelope. If this test results were abnormal, I would be offered a colonoscopy. | |
| **Received by all participants via the booklet:** | | | | | |
|  | . A colonoscopy is an investigation that involves looking directly at the lining of your large bowel. A thin, flexible tube with a tiny camera attached (a colonoscope) is passed into your back passage and guided around your bowel. If polyps are found, most can be removed painlessly, using a wire loop passed down the colonoscopy tube. These tissue samples would be checked for any abnormal cells that might be cancerous.  . A colonoscopy is the most effective way to diagnose bowel polyps and cancer. For most people having a colonoscopy is a straightforward procedure. However, as with most medical procedures, there is the possibility of complications. These can include heavy bleeding (about a one in 150 chance) that needs further investigation or medical advice. The colonoscopy can cause a hole (perforation) in the wall of the bowel (about a one in 1,500 chance). In extremely rare cases, colonoscopy may result in death. Current evidence suggests that this may only happen in about one in 10,000 cases.  . If a polyp is found, you might be offered a follow-up test called a colonoscopy. Removing polyps during a colonoscopy can prevent bowel cancer. | | | |  |
| **Received by participants randomised to colonoscopy only (participants randomised to other test conditions received information on similar concepts):** | | | | | |
| *Benefits* |  | | | |  |
|  |  | | | |  |
| **Aims** | . Aims to detect pre-cancerous polyps, which can be removed to prevent cancer  . Aims to detect cancer at an early stage when it is more treatable | | | |  |
| **Incidence and mortality reduction** | . Estimated to reduce the risk of getting bowel cancer by **at least 23%** and dying from bowel cancer by **at least 31%** | | | |  |
| *Practicalities* |  | | | |  |
|  |  | | | |  |
| **Test frequency and eligible age** | . A one-off test at 55 years | | | |  |
|  |  | | | |  |
| **Overview** | . Looks inside the full length of the large bowel for polyps or cancers  . The test can remove polyps and take samples of cancer | | | |  |
|  |  | | | |  |
|  |  | | | |  |
| **Preparation and procedure** | . A powerful laxative preparation would need to be drunk (causing diarrhoea) and dietary restrictions followed at home to clean out the bowel before the test  . The test would be carried out in hospital by a specialist  . A person would be given a muscle relaxant, painkiller and sedative that would make a person drowsy with effects lasting up to 24 hours after the test  . A thin, flexible tube with a camera on the end would be passed through the large bowel to look for polyps or cancers | | | |  |
|  |  | | | |  |
| **Duration** | 30-45 minutes | | | |  |
|  |  | | | |  |
|  |  | | | |  |
| **Risks** | . Not being able to see all of the large bowel, which may require another test  . Perforation in **one in 1,500** tests, which may require an operation  . Heavy bleeding in **one in 150** tests, which may require further investigation or medical advice  . Breathing or heart problems if people have a reaction to the sedative (rare)  . Death in extremely rare cases (one in 10,000) | | | |  |
|  |  | | | |  |
|  |  | | | |  |
| **Results delivery** | . Immediately after the test; biopsy results within three weeks | | | |  |
|  |  | | | |  |
| **Results of the screening test** | . A normal result in **675 in 1,000** people who would not need any further tests  . One or more polyps found in **320 in 1,000** people. If a polyp is removed, a person would be told whether they are in a low, intermediate or high risk group. People in intermediate or high risk groups would be offered a colonoscopy every one or three years  . A cancer found in **5 in 1,000** people who would be referred for treatment but may have a colonoscopy first | | | |  |
|  |  | | | |  |
| **Sensitivity for cancers and polyps** | . Over **90% accurate** for detecting polyps or bowel cancer but there is a chance that a polyp or cancer could be missed | | | |  |
|  |  | | | |  |
| **Results of any follow-up tests** | . No follow-up investigations apply | | | |  |
