# Supplementary figures and images for: Using a hypothetical scenario to assess public preferences for colorectal surveillance following screening-detected, intermediate-risk adenomas: annual home-based stool test vs. triennial colonoscopy
Source: BMC Gastroenterol. 2016 Sep 13;16(1):113. doi: 10.1186/s12876-016-0517-1 (PMC5020544; doi:10.1186/s12876-016-0517-1)

**Additional file 2 - Items relating to surveillance on each of the questionnaires**


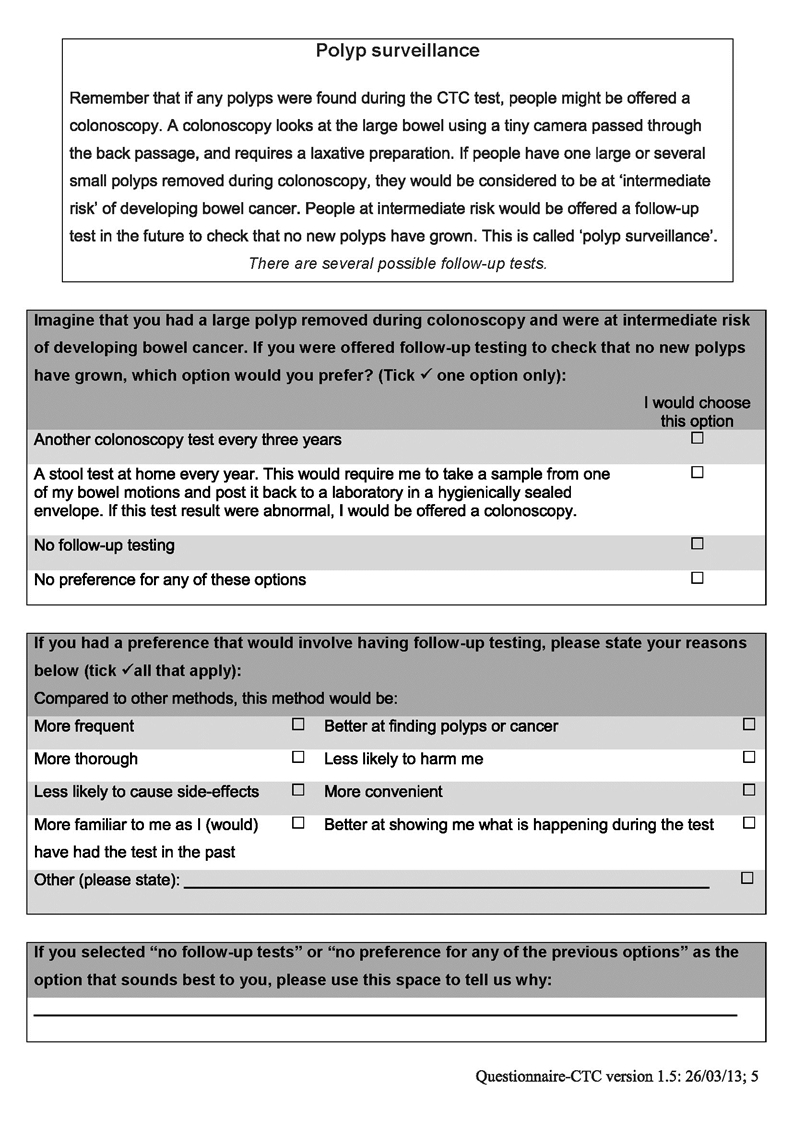

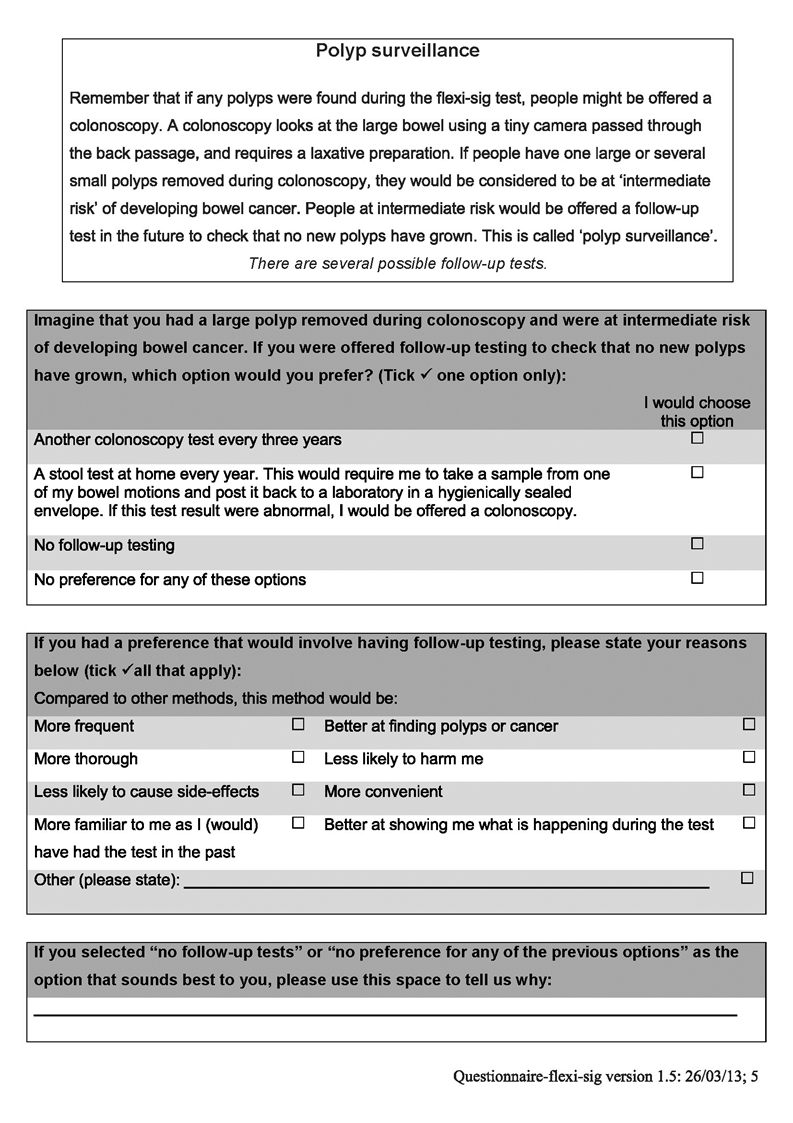

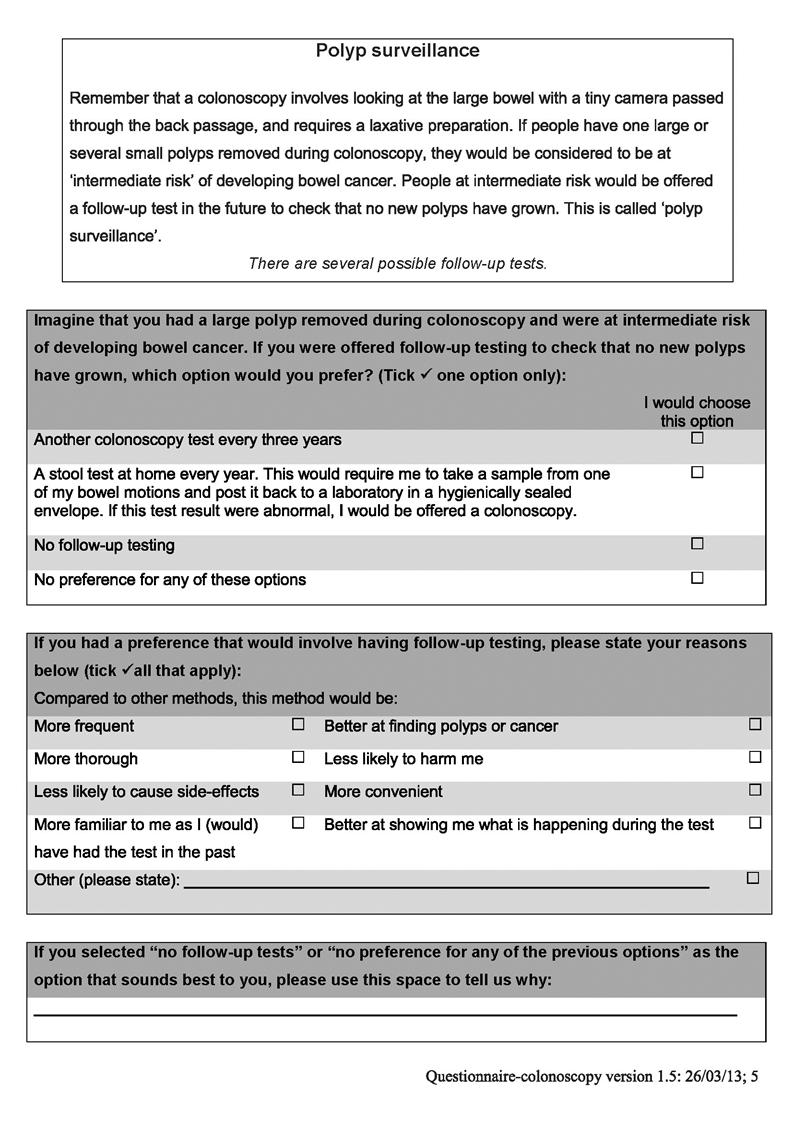

Supplement: Additional file 2: — Questionnaire items relating to surveillance. (DOCX 1099 kb) [file 12876_2016_517_MOESM2_ESM.docx]
